# Diaqua­(5-methyl-1*H*-pyrazole-3-carboxyl­ato)(4-nitro­benzoato)copper(II)

**DOI:** 10.1107/S160053680900169X

**Published:** 2009-01-23

**Authors:** Fei-long Hu, Xian-hong Yin, Yu Feng, Yan Mi, Shan-shan Zhang

**Affiliations:** a College of Chemistry and Ecological Engineering, Guangxi University for Nationalities, Nanning 530006, People’s Republic of China

## Abstract

In the title complex, [Cu(C_7_H_4_NO_4_)(C_5_H_5_N_2_O_2_)(H_2_O)_2_], the Cu^II^ ion is coordinated in a slightly distorted square-pyramidal enviroment. The basal plane is formed by an N atom and an O atom from a 5-methyl-1*H*-pyrazole-3-carboxyl­ate ligand and by two O atoms from two water ligands. The apical position is occupied by a carboxylate O atom from a 4-nitro­benzoate ligand. In the crystal structure, inter­molecular O—H⋯O and N—H⋯O hydrogen bonds link complex moleclues, forming extended chains parallel to the *a* axis.

## Related literature

For background information, see: Montoya *et al.* (2007 ▶).
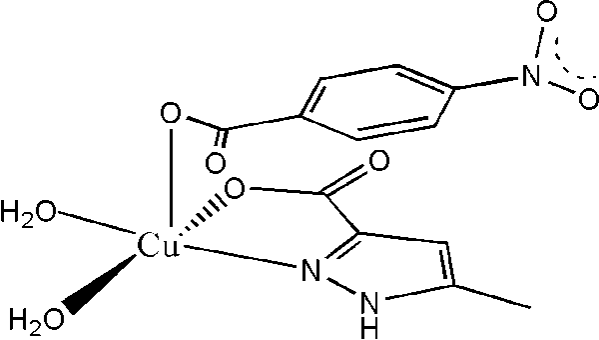


## Experimental

### 

#### Crystal data

[Cu(C_7_H_4_NO_4_)(C_5_H_5_N_2_O_2_)(H_2_O)_2_]
*M* *_r_* = 390.79
Triclinic, 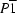

*a* = 6.965 (1) Å
*b* = 9.1860 (13) Å
*c* = 12.4220 (16) Å
α = 96.633 (1)°
β = 105.116 (2)°
γ = 103.978 (2)°
*V* = 730.91 (17) Å^3^
*Z* = 2
Mo *K*α radiation
μ = 1.55 mm^−1^
*T* = 298 (2) K
0.40 × 0.21 × 0.20 mm

#### Data collection

Siemens SMART CCD diffractometer
Absorption correction: multi-scan (*SADABS*; Sheldrick, 1996 ▶) *T*_min_ = 0.577, *T*_max_ = 0.748
3783 measured reflections
2526 independent reflections
2249 reflections with *I* > 2σ(*I*)
*R*_int_ = 0.013

#### Refinement

*R*[*F* ^2^ > 2σ(*F* ^2^)] = 0.028
*wR*(*F* ^2^) = 0.073
*S* = 1.04
2526 reflections
218 parameters
H-atom parameters constrained
Δρ_max_ = 0.39 e Å^−3^
Δρ_min_ = −0.32 e Å^−3^

### 

Data collection: *SMART* (Siemens, 1996 ▶); cell refinement: *SAINT* (Siemens, 1996 ▶); data reduction: *SAINT*; program(s) used to solve structure: *SHELXS97* (Sheldrick, 2008 ▶); program(s) used to refine structure: *SHELXL97* (Sheldrick, 2008 ▶); molecular graphics: *DIAMOND* (Brandenburg, 2006 ▶); software used to prepare material for publication: *SHELXTL* (Sheldrick, 2008 ▶).

## Supplementary Material

Crystal structure: contains datablocks I, global. DOI: 10.1107/S160053680900169X/lh2751sup1.cif

Structure factors: contains datablocks I. DOI: 10.1107/S160053680900169X/lh2751Isup2.hkl

Additional supplementary materials:  crystallographic information; 3D view; checkCIF report

## Figures and Tables

**Table 1:** Selected bond lengths (Å)

Cu1—O8	1.9344 (17)
Cu1—O7	1.9489 (17)
Cu1—N1	1.970 (2)
Cu1—O1	1.9811 (16)
Cu1—O3	2.3164 (19)

**Table 2:** Hydrogen-bond geometry (Å, °)

*D*—H⋯*A*	*D*—H	H⋯*A*	*D*⋯*A*	*D*—H⋯*A*
O7—H7*A*⋯O2^i^	0.85	1.96	2.799 (2)	173
O7—H7*B*⋯O3^ii^	0.84	1.83	2.631 (2)	157
O8—H8*A*⋯O1^iii^	0.85	1.97	2.803 (3)	165
O8—H8*B*⋯O4^ii^	0.85	1.78	2.582 (2)	156
N2—H2⋯O2^i^	0.86	1.97	2.781 (3)	157

Symmetry codes: (i) 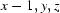; (ii) 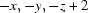; (iii) 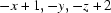.

**Fig. 1.**
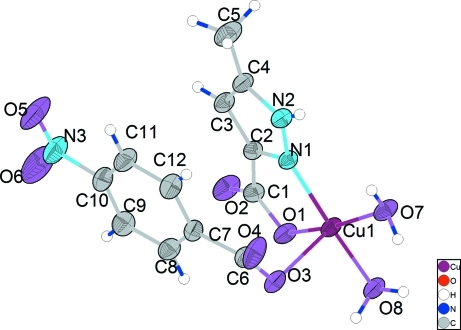
The molecular structure of the title compound (I) showing 50% proability displacement ellipsoids and the atom-numbering scheme.

**Fig. 2.**
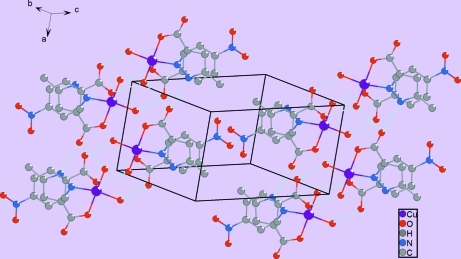
Part of the crystal structure of (I) showing the donor-to-acceptor distances of hydrogen bonds as dashed lines.

**Fig. 3.**
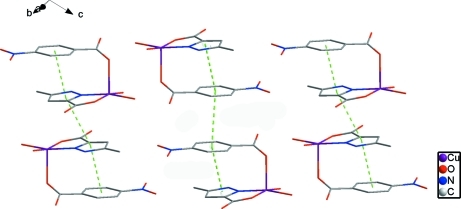
Part of the crystal structure of (I) illustrating π-π stacking interactions as dashed lines.

## supplementary crystallographic information

### Comment

The chemical and pharmacological properties of pyrazoles have been investigated extensively, owing to their chelating ability with metal ions and their potentially beneficial chemical and biological activities (Montoya *et al.*, 2007.) As part of our studies on the synthesis and characterization of these types of compounds, we report here the synthesis and crystal structure of the title compound (I).

The molecular structure of (I) is shown in Fig. 1. The Cu atom is five-coordinated by four O atoms and one N atom. The basal plane is formed by an N atom and an O atom from a 5-methyl-1*H*-pyrazole-3-carboxylato ligand and two O atoms from coordinated water molecules. In the crystal structure, intermolecular O—H···O and N—H···O hydrogen bonds (Fig. 2) link complex molecules, to form extended chains parallel to the *a* axis. The coordinated water molecules act as hydrogen donors for symmetry related carboxyl O atoms (see Table 2). In addition, the crystal structure contains various π-π stacking interactions involving the C7-C12, N1/N2/C2-C4 and Cu1/O1/C1/C2/N1 rings with a range of centroid-to-centroid distances of 3.265 (1)-3.849 (1)Å (see Fig. 3).

### Experimental

5-methyl-1*H*-pyrazole-3-carboxylic acid,4-nitrobenzoic acid and CuCl_2_.6H_2_O were available commercially and were used without further purification. Equimolar amounts of 5-methyl-1*H*-pyrazole-3-carboxylic acid (0.5 mmol, 63.02 mg) and 4-nitrobenzoic acid (0.5 mmol, 83.51 mg) were dissolved in anhydrous alcohol (15 ml). The mixture was stirred to give a clear solution, to this solution was added CuCl_2_.6H_2_O (0.5 mmol, 113 mg) in anhydrous alcohol (10 ml). After keeping the resulting solution in air to evaporate about half of the solvents, blue prisms of the title compound were formed. The crystals were isolated, washed with alcohol three times and dried in a vacuum desiccator using silica gel (Yield 75%). Elemental analysis: found: C, 36.82; H, 3.38; N, 10.65%. calc. for C_12_H_13_CuN_3_O_8_: C, 36.88; H, 3.35; N, 10.75%.

### Refinement

H atoms attached to C amd N atoms were positioned geometrically and refined using a riding-model approximation with C–H = 0.93–0.96 Å; N-H = 0.86Å and *U*_iso_(H) = 1.2–1.5*U*_eq_(C, N). The water H atoms were located in difference Fourier maps and included in 'as found' positions in a riding-model approximation with *U*_iso_(H) = 1.5*U*_eq_(O).

### Crystal data

**Table:**

[Cu(C_7_H_4_NO_4_)(C_5_H_5_N_2_O_2_)(H_2_O)_2_]	*Z* = 2
*M**_r_* = 390.79	*F*(000) = 398
Triclinic, *P*1	*D*_x_ = 1.776 Mg m^−^^3^
Hall symbol: -P 1	Mo *K*α radiation, λ = 0.71073 Å
*a* = 6.965 (1) Å	Cell parameters from 2564 reflections
*b* = 9.1860 (13) Å	θ = 2.6–27.9°
*c* = 12.4220 (16) Å	µ = 1.55 mm^−^^1^
α = 96.633 (1)°	*T* = 298 K
β = 105.116 (2)°	Block, blue
γ = 103.978 (2)°	0.40 × 0.21 × 0.20 mm
*V* = 730.91 (17) Å^3^	

### Data collection

**Table:**

Siemens SMART CCD diffractometer	2526 independent reflections
Radiation source: fine-focus sealed tube	2249 reflections with *I* > 2σ(*I*)
graphite	*R*_int_ = 0.013
φ and ω scans	θ_max_ = 25.0°, θ_min_ = 1.7°
Absorption correction: multi-scan (*SADABS*; Sheldrick, 1996)	*h* = −8→8
*T*_min_ = 0.577, *T*_max_ = 0.748	*k* = −10→10
3783 measured reflections	*l* = −10→14

### Refinement

**Table:**

Refinement on *F*^2^	Primary atom site location: structure-invariant direct methods
Least-squares matrix: full	Secondary atom site location: difference Fourier map
*R*[*F*^2^ > 2σ(*F*^2^)] = 0.028	Hydrogen site location: inferred from neighbouring sites
*wR*(*F*^2^) = 0.073	H-atom parameters constrained
*S* = 1.04	*w* = 1/[σ^2^(*F*_o_^2^) + (0.0335*P*)^2^ + 0.6897*P*] where *P* = (*F*_o_^2^ + 2*F*_c_^2^)/3
2526 reflections	(Δ/σ)_max_ = 0.001
218 parameters	Δρ_max_ = 0.39 e Å^−^^3^
0 restraints	Δρ_min_ = −0.32 e Å^−^^3^

### Special details

**Table:**

Geometry. All e.s.d.'s (except the e.s.d. in the dihedral angle between two l.s. planes) are estimated using the full covariance matrix. The cell e.s.d.'s are taken into account individually in the estimation of e.s.d.'s in distances, angles and torsion angles; correlations between e.s.d.'s in cell parameters are only used when they are defined by crystal symmetry. An approximate (isotropic) treatment of cell e.s.d.'s is used for estimating e.s.d.'s involving l.s. planes.
Refinement. Refinement of *F*^2^ against ALL reflections. The weighted *R*-factor *wR* and goodness of fit *S* are based on *F*^2^, conventional *R*-factors *R* are based on *F*, with *F* set to zero for negative *F*^2^. The threshold expression of *F*^2^ > σ(*F*^2^) is used only for calculating *R*-factors(gt) *etc*. and is not relevant to the choice of reflections for refinement. *R*-factors based on *F*^2^ are statistically about twice as large as those based on *F*, and *R*- factors based on ALL data will be even larger.

### Fractional atomic coordinates and isotropic or equivalent isotropic displacement parameters (Å^2^)

**Table:**

	*x*	*y*	*z*	*U*_iso_*/*U*_eq_	
Cu1	0.29638 (4)	0.19451 (3)	0.99760 (3)	0.02357 (11)	
O1	0.5665 (3)	0.2069 (2)	0.96944 (15)	0.0277 (4)	
O2	0.7980 (3)	0.3422 (2)	0.89863 (17)	0.0381 (5)	
O3	0.1131 (3)	−0.0129 (2)	0.85168 (15)	0.0304 (4)	
O4	−0.1882 (3)	−0.0059 (3)	0.73675 (17)	0.0436 (5)	
O5	0.3529 (4)	0.3559 (3)	0.4025 (2)	0.0652 (7)	
O6	0.6072 (4)	0.2702 (4)	0.4743 (3)	0.0742 (9)	
O7	0.0648 (3)	0.2226 (2)	1.04985 (15)	0.0300 (4)	
H7A	−0.0183	0.2515	0.9997	0.045*	
H7B	0.0013	0.1397	1.0639	0.045*	
O8	0.3635 (3)	0.0686 (2)	1.10838 (15)	0.0307 (4)	
H8A	0.3628	−0.0188	1.0768	0.046*	
H8B	0.2798	0.0576	1.1478	0.046*	
N1	0.2972 (3)	0.3507 (2)	0.90140 (17)	0.0220 (4)	
N2	0.1750 (3)	0.4334 (2)	0.85329 (17)	0.0240 (4)	
H2	0.0547	0.4290	0.8609	0.029*	
N3	0.4354 (4)	0.2843 (3)	0.4672 (2)	0.0445 (6)	
C1	0.6256 (4)	0.3104 (3)	0.9141 (2)	0.0240 (5)	
C2	0.4692 (3)	0.3913 (3)	0.8697 (2)	0.0212 (5)	
C3	0.4564 (4)	0.5001 (3)	0.8016 (2)	0.0265 (5)	
H3	0.5558	0.5468	0.7693	0.032*	
C4	0.2652 (4)	0.5246 (3)	0.7917 (2)	0.0265 (5)	
C5	0.1576 (5)	0.6251 (4)	0.7292 (3)	0.0449 (8)	
H5A	0.0132	0.5718	0.6957	0.067*	
H5B	0.2181	0.6526	0.6707	0.067*	
H5C	0.1721	0.7159	0.7811	0.067*	
C6	0.0028 (4)	0.0150 (3)	0.7621 (2)	0.0272 (6)	
C7	0.1138 (4)	0.0802 (3)	0.6810 (2)	0.0252 (5)	
C8	0.3027 (4)	0.0557 (3)	0.6794 (2)	0.0293 (6)	
H8	0.3590	−0.0045	0.7267	0.035*	
C9	0.4072 (4)	0.1196 (3)	0.6083 (2)	0.0321 (6)	
H9	0.5324	0.1022	0.6064	0.038*	
C10	0.3217 (4)	0.2099 (3)	0.5403 (2)	0.0311 (6)	
C11	0.1344 (4)	0.2360 (3)	0.5388 (2)	0.0344 (6)	
H11	0.0796	0.2969	0.4916	0.041*	
C12	0.0298 (4)	0.1692 (3)	0.6095 (2)	0.0318 (6)	
H12	−0.0978	0.1841	0.6090	0.038*	

### Atomic displacement parameters (Å^2^)

**Table:**

	*U*^11^	*U*^22^	*U*^33^	*U*^12^	*U*^13^	*U*^23^
Cu1	0.01987 (17)	0.02766 (19)	0.02970 (19)	0.00910 (12)	0.01187 (13)	0.01551 (13)
O1	0.0231 (9)	0.0327 (10)	0.0370 (10)	0.0137 (8)	0.0148 (8)	0.0191 (8)
O2	0.0228 (10)	0.0500 (13)	0.0556 (13)	0.0161 (9)	0.0218 (9)	0.0290 (10)
O3	0.0326 (10)	0.0323 (10)	0.0270 (10)	0.0051 (8)	0.0103 (8)	0.0141 (8)
O4	0.0300 (11)	0.0721 (15)	0.0385 (11)	0.0153 (10)	0.0180 (9)	0.0289 (11)
O5	0.0705 (17)	0.0853 (19)	0.0645 (16)	0.0304 (15)	0.0368 (14)	0.0546 (15)
O6	0.0562 (16)	0.110 (2)	0.095 (2)	0.0382 (16)	0.0546 (15)	0.0648 (18)
O7	0.0239 (9)	0.0356 (11)	0.0418 (11)	0.0134 (8)	0.0176 (8)	0.0230 (9)
O8	0.0342 (10)	0.0357 (10)	0.0351 (10)	0.0173 (8)	0.0194 (8)	0.0204 (8)
N1	0.0207 (10)	0.0243 (11)	0.0259 (11)	0.0088 (8)	0.0108 (8)	0.0102 (9)
N2	0.0195 (10)	0.0278 (11)	0.0317 (11)	0.0115 (9)	0.0122 (9)	0.0128 (9)
N3	0.0464 (16)	0.0533 (17)	0.0445 (15)	0.0145 (13)	0.0242 (12)	0.0257 (13)
C1	0.0205 (12)	0.0245 (13)	0.0291 (13)	0.0067 (10)	0.0094 (10)	0.0086 (10)
C2	0.0180 (12)	0.0211 (12)	0.0262 (13)	0.0036 (9)	0.0102 (10)	0.0067 (10)
C3	0.0255 (13)	0.0275 (14)	0.0326 (14)	0.0071 (11)	0.0164 (11)	0.0127 (11)
C4	0.0294 (14)	0.0265 (14)	0.0295 (14)	0.0102 (11)	0.0134 (11)	0.0132 (11)
C5	0.0460 (18)	0.053 (2)	0.0553 (19)	0.0281 (15)	0.0255 (15)	0.0348 (16)
C6	0.0290 (14)	0.0264 (14)	0.0271 (14)	0.0052 (11)	0.0116 (11)	0.0070 (11)
C7	0.0250 (13)	0.0268 (14)	0.0221 (13)	0.0038 (10)	0.0072 (10)	0.0054 (10)
C8	0.0283 (14)	0.0326 (15)	0.0299 (14)	0.0112 (11)	0.0084 (11)	0.0121 (11)
C9	0.0248 (13)	0.0395 (16)	0.0357 (15)	0.0108 (12)	0.0121 (11)	0.0109 (12)
C10	0.0299 (14)	0.0369 (15)	0.0290 (14)	0.0058 (12)	0.0143 (11)	0.0110 (11)
C11	0.0371 (15)	0.0409 (17)	0.0318 (15)	0.0165 (13)	0.0112 (12)	0.0190 (12)
C12	0.0250 (13)	0.0440 (17)	0.0312 (14)	0.0136 (12)	0.0099 (11)	0.0142 (12)

### Geometric parameters (Å, °)

**Table:**

Cu1—O8	1.9344 (17)	C1—C2	1.488 (3)
Cu1—O7	1.9489 (17)	C2—C3	1.387 (3)
Cu1—N1	1.970 (2)	C3—C4	1.380 (3)
Cu1—O1	1.9811 (16)	C3—H3	0.9300
Cu1—O3	2.3164 (19)	C4—C5	1.488 (4)
O1—C1	1.286 (3)	C5—H5A	0.9600
O2—C1	1.236 (3)	C5—H5B	0.9600
O3—C6	1.269 (3)	C5—H5C	0.9600
O4—C6	1.244 (3)	C6—C7	1.515 (3)
O5—N3	1.213 (3)	C7—C12	1.388 (4)
O6—N3	1.216 (3)	C7—C8	1.392 (4)
O7—H7A	0.8468	C8—C9	1.379 (4)
O7—H7B	0.8446	C8—H8	0.9300
O8—H8A	0.8503	C9—C10	1.376 (4)
O8—H8B	0.8496	C9—H9	0.9300
N1—C2	1.339 (3)	C10—C11	1.378 (4)
N1—N2	1.345 (3)	C11—C12	1.386 (4)
N2—C4	1.352 (3)	C11—H11	0.9300
N2—H2	0.8599	C12—H12	0.9300
N3—C10	1.475 (3)		
			
O8—Cu1—O7	91.42 (7)	C4—C3—C2	105.6 (2)
O8—Cu1—N1	166.43 (8)	C4—C3—H3	127.2
O7—Cu1—N1	96.45 (7)	C2—C3—H3	127.2
O8—Cu1—O1	88.78 (7)	N2—C4—C3	106.6 (2)
O7—Cu1—O1	167.50 (8)	N2—C4—C5	121.2 (2)
N1—Cu1—O1	81.15 (7)	C3—C4—C5	132.2 (2)
O8—Cu1—O3	93.71 (7)	C4—C5—H5A	109.5
O7—Cu1—O3	97.90 (7)	C4—C5—H5B	109.5
N1—Cu1—O3	96.12 (7)	H5A—C5—H5B	109.5
O1—Cu1—O3	94.56 (7)	C4—C5—H5C	109.5
C1—O1—Cu1	115.62 (15)	H5A—C5—H5C	109.5
C6—O3—Cu1	116.46 (16)	H5B—C5—H5C	109.5
Cu1—O7—H7A	110.3	O4—C6—O3	125.0 (2)
Cu1—O7—H7B	109.9	O4—C6—C7	118.0 (2)
H7A—O7—H7B	109.5	O3—C6—C7	117.0 (2)
Cu1—O8—H8A	111.4	C12—C7—C8	119.3 (2)
Cu1—O8—H8B	111.3	C12—C7—C6	119.8 (2)
H8A—O8—H8B	109.4	C8—C7—C6	120.8 (2)
C2—N1—N2	105.59 (19)	C9—C8—C7	120.7 (2)
C2—N1—Cu1	114.33 (15)	C9—C8—H8	119.6
N2—N1—Cu1	140.08 (16)	C7—C8—H8	119.6
N1—N2—C4	111.61 (19)	C10—C9—C8	118.4 (2)
N1—N2—H2	124.2	C10—C9—H9	120.8
C4—N2—H2	124.2	C8—C9—H9	120.8
O5—N3—O6	123.2 (3)	C9—C10—C11	122.6 (2)
O5—N3—C10	118.5 (2)	C9—C10—N3	119.2 (2)
O6—N3—C10	118.3 (2)	C11—C10—N3	118.2 (2)
O2—C1—O1	123.6 (2)	C10—C11—C12	118.3 (2)
O2—C1—C2	121.8 (2)	C10—C11—H11	120.9
O1—C1—C2	114.6 (2)	C12—C11—H11	120.9
N1—C2—C3	110.7 (2)	C11—C12—C7	120.6 (2)
N1—C2—C1	114.0 (2)	C11—C12—H12	119.7
C3—C2—C1	135.4 (2)	C7—C12—H12	119.7
			
O8—Cu1—O1—C1	−165.77 (18)	O1—C1—C2—C3	−175.1 (3)
O7—Cu1—O1—C1	−74.7 (4)	N1—C2—C3—C4	−0.4 (3)
N1—Cu1—O1—C1	5.10 (18)	C1—C2—C3—C4	179.1 (3)
O3—Cu1—O1—C1	100.61 (18)	N1—N2—C4—C3	−0.5 (3)
O8—Cu1—O3—C6	167.18 (17)	N1—N2—C4—C5	179.1 (2)
O7—Cu1—O3—C6	75.23 (18)	C2—C3—C4—N2	0.6 (3)
N1—Cu1—O3—C6	−22.18 (18)	C2—C3—C4—C5	−179.1 (3)
O1—Cu1—O3—C6	−103.75 (17)	Cu1—O3—C6—O4	−100.5 (3)
O8—Cu1—N1—C2	40.1 (4)	Cu1—O3—C6—C7	78.9 (2)
O7—Cu1—N1—C2	165.23 (17)	O4—C6—C7—C12	25.6 (4)
O1—Cu1—N1—C2	−2.40 (17)	O3—C6—C7—C12	−153.8 (2)
O3—Cu1—N1—C2	−96.07 (17)	O4—C6—C7—C8	−156.2 (3)
O8—Cu1—N1—N2	−140.9 (3)	O3—C6—C7—C8	24.3 (4)
O7—Cu1—N1—N2	−15.8 (3)	C12—C7—C8—C9	0.5 (4)
O1—Cu1—N1—N2	176.6 (3)	C6—C7—C8—C9	−177.7 (2)
O3—Cu1—N1—N2	82.9 (3)	C7—C8—C9—C10	0.9 (4)
C2—N1—N2—C4	0.3 (3)	C8—C9—C10—C11	−1.4 (4)
Cu1—N1—N2—C4	−178.7 (2)	C8—C9—C10—N3	177.5 (3)
Cu1—O1—C1—O2	173.2 (2)	O5—N3—C10—C9	175.5 (3)
Cu1—O1—C1—C2	−6.5 (3)	O6—N3—C10—C9	−4.0 (4)
N2—N1—C2—C3	0.1 (3)	O5—N3—C10—C11	−5.5 (4)
Cu1—N1—C2—C3	179.42 (17)	O6—N3—C10—C11	174.9 (3)
N2—N1—C2—C1	−179.5 (2)	C9—C10—C11—C12	0.4 (4)
Cu1—N1—C2—C1	−0.2 (3)	N3—C10—C11—C12	−178.4 (3)
O2—C1—C2—N1	−175.2 (2)	C10—C11—C12—C7	1.0 (4)
O1—C1—C2—N1	4.5 (3)	C8—C7—C12—C11	−1.4 (4)
O2—C1—C2—C3	5.2 (5)	C6—C7—C12—C11	176.7 (3)

### Hydrogen-bond geometry (Å, °)

**Table:**

*D*—H···*A*	*D*—H	H···*A*	*D*···*A*	*D*—H···*A*
O7—H7A···O2^i^	0.85	1.96	2.799 (2)	173
O7—H7B···O3^ii^	0.84	1.83	2.631 (2)	157
O8—H8A···O1^iii^	0.85	1.97	2.803 (3)	165
O8—H8B···O4^ii^	0.85	1.78	2.582 (2)	156
N2—H2···O2^i^	0.86	1.97	2.781 (3)	157

Symmetry codes: (i) *x*−1, *y*, *z*; (ii) −*x*, −*y*, −*z*+2; (iii) −*x*+1, −*y*, −*z*+2.
